# Gonorrhœal Infection in the Female and Its Treatment

**Published:** 1907-11-09

**Authors:** F. J. McCann


### HOSPITAL CLINICS.

### GONORRHŒAL INFECTION IN THE FEMALE.

By F. J. McCANN, M.D., F.R.C.S.(Eng.), M.R.C.P.

### DIAGNOSIS.

THERE is little difficulty about the diagnosis of acute gonorrhœal infection. It is characterised by the sudden onset of pain, painful and frequent micturition, and œdema of the labia, a discharge which is not glutinous, pain in the loins, and in the hypogastrium. There are certain signs which ought to guide you. In cases where, say, the patient is recently married, has not been previously treated, and comes complaining of acute vaginal discharge, developing suddenly, there can be hardly any doubt about diagnosing gonorrhœal infection. The difficulty is greater where the patient has had several children. In that case, you have to look to local signs for help in diagnosis. Look carefully at the labia, and at Bartholin's glands and their ducts, which open just outside the hymen. You will frequently find that the duct is surrounded by a reddened area which is termed the Macula gonorrhoica—the gonorrhœal spot. This is very characteristic. Sometimes there is a chronic gleety discharge from that duct; if it is present, you can be fairly sure it is a gonorrhœal case. You next proceed to examine the vagina. If there is a vaginal discharge it is very difficult, in married women especially, to detect the gonococcus in the discharge, it is said because the thickness of the vaginal epithelium resists the action of the gonococcus. That is true to a certain extent, but you will find that you can get the gonococcus in the vaginal discharge of recently married women, where the vaginal epithelium is more delicate, and you can get it in the discharge of the vagina in children, and in that of pregnant women. In other cases it is not found.

You will find a marked congestion on the walls of the vagina, a deep red colour with pus on the surface. It is a thin purulent discharge, not the glutinous secretion of the ordinary leucorrhœa; underneath it is a raw bloody surface. Examine the cervix, and in this case it is well for you to take some of the discharge from the surface and examine it. It is a good plan to put in a tampon of salicylic acid over night, remove it in the morning, and so obtain pus for examination. Another important diagnostic is the presence of tubal disease. If you have a case of chronic vaginal discharge, and in addition double tubal disease, the gonococcus is almost certainly the cause of both. Tubercular cases characteristically form elongated masses as compared with those of a banana shape presented in gonorrhœa, when the appendages are matted together.

How is it that gonorrhœa spreads to the tubes? The chief reason is that it has not been properly treated. Apart from that, there are other reasons, the chief of which is sexual intercourse during the disease. Another is treatment by the passing of sounds or probes into the uterus, and a third is pregnancy. That being so, the important point is to kill the gonococcus at as early a date as possible, and yet we seldom find this done. When the patient contracts gonorrhœa she probably remains quiet for the first two days during the acute stage. As soon as that is over she proceeds to use a syringe, and carries infective matter into the uterus, and thence into the tubes. I believe it is douching which is the cause of a great deal of tubal disease following gonorrhœa, and that those who do not get tubal disease are persons who have no treatment at all, and simply let the disease run dry.

This emphasises what I would like to say on the great value of immediate treatment in gonorrhœal cases. The patient should never use a douche, although most text-books advise one twice a day. It is a perpetual reinfection, and frequently causes uterine colic. In the treatment of gonorrhœa, douching should be given up absolutely. Instead of this the treatment should be carried out by the medical man, who should locally swab the diseased surface. Hot sitz and other baths should be avoided. There can be nothing worse than passing solid caustics along the urethra. They frequently give rise to stricture, but it is not at all an uncommon method of treating gonorrhœa. In any method of treatment you adopt, if you cannot do the patient any good, do not do her any harm.

Cystitis may be troublesome and frequently becomes chronic. The more I see of its treatment the more I am convinced that the great value of any washing out of the bladder is a purely mechanical one, and in most cases I wash out the bladder with plain boiled water. A large number of antiseptics or germicides are used for this purpose, and some are used in much too strong solutions. You will get quite as good results if you wash with weak boric lotion or plain boiled water. Lately we have had one or two good drugs in the shape of urinary antiseptics. Urotropine, cystamine, and helmitol are the three best. Sometimes urotropine does not act, and then one tries cystamine or helmitol, five or six grains to the dose.

### VULVITIS AND CATARRH OF THE BARTHOLINIAN DUCTS.

Chronic gleet of this duct is often to be seen, if looked for. It is also common to see an unfortunate patient being told to douche herself three or four times a day, although the seat of the discharge is the Bartholinian ducts. The best treatment for that condition is to slit up the duct with a canaliculus knife, when the condition will soon clear up. If abscess formation takes place in the ducts or in the glands, it should be remembered that all these cases are not gonorrhœal, although some text-books say they are. Some are due to injury to the mouth of the duct, and septic invasion by staphylococci or streptococci. A certain number are gonorrhœal, and these sometimes burst and burrow in the labia and cause sinuses in different positions there. When you have such a condition as that you require to dissect out the sinuses and the abscess in the duct, and bring the incision carefully together. The incision must be free (no little cuts are suitable in such cases) and plugged with gauze until it heals up from below. Any number of these cases are allowed to go on discharging, which could be healed up in a few days. Infection of the opposite side has to be avoided in treating this Bartholinian abscess. Another practical point is the treatment of gonorrhœal warts. These may be arranged like buds, and the best treatment is to snip them off with the scissors, and touch the beds either with nitric acid or with a cautery. Powders and lotions are of no use in these cases.

Pruritis developed in consequence of gonorrhœa is a very persistent and troublesome condition. When you get persistent pruritis in a relatively young woman, be suspicious that the case is one of gonorrhœa. The inflamed surface should be protected from the discharge by lanoline, vaseline, or bismuth. The latter is excellent, not only as a powder, but also combined with lanoline for irritation in this region.

### Vaginitis and Endo-cervicitis.

Suppose we are dealing with a case of acute gonorrhœa. Put the patient to bed, and keep her there; insist on the avoidance of all wines, spirits, beers, and on a supply of diluents. The bowels should be freely opened by a saline aperient, and if there is much pain a morphia suppository may give relief. The discharge is to be removed by bathing in a hot boracic bath and using cotton wool to clean the vulva, and not a sponge. This acute stage generally lasts about three days. After that is over proceed to kill the poison. The patient will not permit you to do that until the first stage is over. This is done by swabbing out the vagina with strong carbolic through a Fergusson's speculum; by that method you kill the gonococcus, and I will undertake to say that you will cure within four weeks every case of gonorrhœa you have. The acid used is the acidum carbolicum liquefactum of the B.P., not the solid carbolic acid liquefied, which is just a little too strong; being a corrosive poison it must be used with intelligence, or sloughing may be produced. You simply want to sear the surface. You get a Fergusson's speculum and take a bit of wool to protect the anal region, then you take a long pair of forceps and dip a swab into the carbolic, and with another piece of wool remove the excess of carbolic. Pass this through the speculum, and sear the vaginal surfaces, so that it turns white. You are to carefully mop up any excess. If you stop applying the carbolic just as you come to the vulvar orifice no pain is caused. I saw the other day some criticism of this immediate treatment on the ground that it produces too much swelling. If you use carbolic or any other corrosive poison, you may make the patient worse, but that is your fault and not the fault of the method. You have only to sear the surface. If swelling is produced your carbolic has been too strong, or too freely used. To ensure that no excess trickles into the vagina it is useful to insert double cyanide gauze, simply to keep the vaginal walls apart. In a bad case you may have to make the application twice a week. Once may be sufficient, but here let me say that hard and fast rules are of no use. If the application has produced much swelling leave ill alone until the swelling has subsided, and then repeat the treatment. Considering how easily the poison can be reached in the female by means of a speculum, it is astonishing that this method is not more universally adopted. As a rule the patient is told to syringe, and left to treat herself. The result is that she gets worse and worse, and this accounts for the number of tubal cases one sees.

Some men use corrosive sublimate either in the strength of one in a thousand or one in five hundred, but you must remember all the time that it is a potent poison, and all excess should be mopped out of the vagina.

Rest is very important in the treatment of these patients, but they belong to that class that refuse to follow directions. As the condition gets better it is a good plan to use for future applications equal parts of glycerine and carbolic, and in addition to pass a pessary into the vagina consisting of oxychloride of bismuth, gr. 10, ol. theobrom, dr. 2. It is allowed to dissolve while the patient is in bed.

In addition to swabbing out the vagina you may also wash out the cervix, but never insert a Playfair's probe further than an inch, and you should do nothing to the cavity or to the body of the uterus. If you curette the patient salpingitis will develop. Local injury seems to stir up the poison in an extraordinary manner. In tubal trouble do everything you can to avoid operation in these gonorrhœal cases. It has now been conclusively shown that such tubes and ovaries can recover under treatment. What treatment can you give? Iodide of potassium seems to have wonderful effects in gonorrhœal cases, or small doses of mercury.

Another necessary is sexual rest—the most difficult of all problems to deal with in practice—the domestic problem. That is why you put the poor in the hospitals, and the rich in the foreign watering places, in order that the patient may get sexual rest. In many of these cases there is a continuous reinfection, and it is important to have both husband and wife under treatment. Six weeks' or two months' sexual abstinence is necessary if the treatment is not to be worthless. Many cases go from bad to worse, because they do not get sexual rest.

In addition, counter irritation, external and internal—internally by painting the fornices with tincture of iodine is the best treatment; externally with equal parts of tincture of iodine and lanoline, or various other counter irritants. With regard to douches, if you give hot douches in certain early cases the patient will probably get pyo-salpingitis, so that you must be very careful in discriminating what state the salpingitis is in. If it is in an early stage it will tend to suppuration. If it is in the chronic adhesive state, when the risk of suppuration is passed, hot douchings do good. You must be careful to be certain that you have appreciated the stage at which the tubal disease has arrived. Douching may rupture a pyosalpinx and cause death.

